# Boredom and curiosity: the hunger and the appetite for information

**DOI:** 10.3389/fpsyg.2024.1514348

**Published:** 2024-12-11

**Authors:** Johannes P.-H. Seiler, Ohad Dan

**Affiliations:** ^1^Focus Program Translational Neurosciences, Institute of Physiology, University Medical Center of the Johannes Gutenberg University Mainz, Mainz, Germany; ^2^Department of Comparative Medicine, Yale School of Medicine, New Haven, CT, United States

**Keywords:** boredom, curiosity, information, information-seeking, exploration, exploration and exploitation

## Abstract

Boredom and curiosity are common everyday states that drive individuals to seek information. Due to their functional relatedness, it is not trivial to distinguish whether an action, for instance in the context of a behavioral experiment, is driven by boredom or curiosity. Are the two constructs opposite poles of the same cognitive mechanism, or distinct states? How do they interact? Can they co-exist and complement each other? Here, we systematically review similarities and dissimilarities of boredom and curiosity with respect to their subjective experience, functional role, and neurocognitive implementation. We highlight the usefulness of Information Theory for formalizing information-seeking in the context of both states and provide guidelines for their experimental investigation. Our emerging view is that despite their distinction on an experiential level, boredom and curiosity are closely related on a functional level, providing complementary drives on information-seeking: boredom, similar to hunger, arises from a lack of information and drives individuals to avoid contexts with low information yield, whereas curiosity constitutes a mechanism similar to appetite, pulling individuals toward specific sources of information. We discuss predictions arising from this perspective, concluding that boredom and curiosity are independent, but coalesce to optimize behavior in environments providing varying levels of information.

## Introduction

*Boredom* and *curiosity* describe familiar, everyday mental states whose cognitive and neural basis receives growing attention from the scientific community. While both terms are broadly defined, there is a substantial overlap between the two with respect to their causal role in information seeking. How separable are the two states? Consider for example a patient in a crowded waiting room who idly scrolls through a magazine's pages, following an intriguing headline on a magazine cover. Is this information-seeking action driven by curiosity or boredom? How can the two be differentiated? Can the two coexist? Or are they, either empirically or by definition, mutually exclusive? Intuitively, “being bored” and “being curious” represent opposite extremes of a single axis that captures engagement in information-seeking behavior. To what extent is this perception consistent with current literature, and if so, what precisely is the relevant axis on which the two concepts oppose each other?

Here, we survey recent literature, contrasting the definitions, operationalization, and experiential implications for boredom and curiosity. The emerging picture offers an integrative framework that informs both the conceptualization and assessment of the two constructs. With these insights, we provide recommendations for future studies, highlighting the potential and pitfalls of studying these phenomena in humans and other animals. We also discuss the present-day relevance of both phenomena as mental health parameters, and future directions for cognitive and neuroscientific research.

## Definitions, overlaps and distinctions of boredom and curiosity

**Figure 1:**
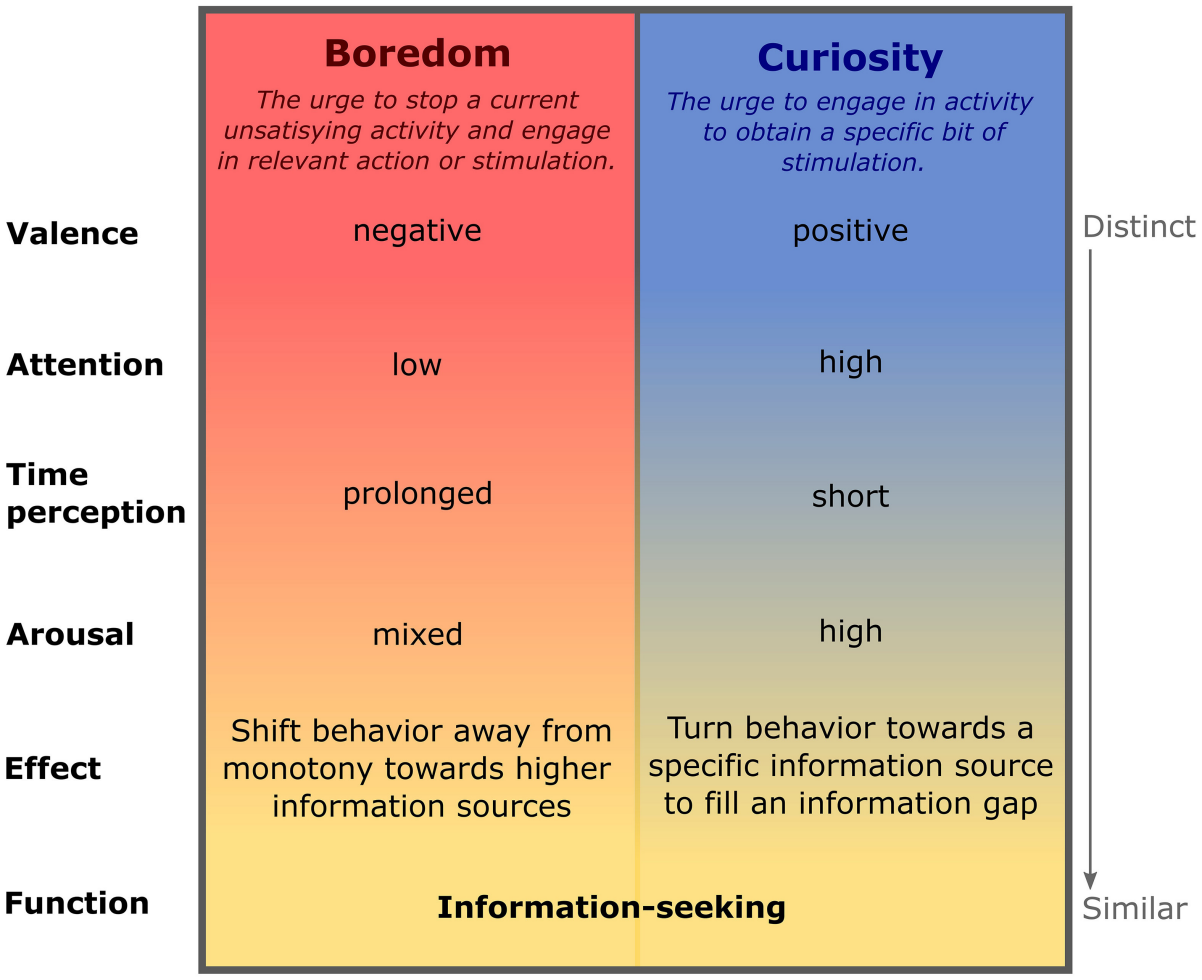
Experiential characteristics of boredom and curiosity: comparison of boredom and curiosity with respect to various dimensions. While valence, attention and time perception of both states is substantially distinct, the behavioral consequences of both phenomena largely overlap, by promoting information-seeking. A detailed description of boredom and curiosity in all dimensions listed in the figure, and the according references can be found in the main text.

Individuals engage with their environment and actively explore it. This fundamental behavioral tendency has widely been observed across different species and experimental contexts (Cloninger et al., 1993; Roberts et al., 2012). For instance, already Ivan Pavlov, one of the fathers of behavioral neuroscience, described that the dogs he used for experiments, strongly reacted to novel and unexpected stimuli by turning their heads and ears toward them in order to actively maximize the incoming information (Pavlov, 1927). Classically, this trend to seek novel information has been attributed to curiosity (Gottlieb et al., 2013; Kidd and Hayden, 2015). However, effective information-seeking cannot only be implemented by seeking the new, it also requires a drive to avoid the old. Especially, in environments that do not provide any interesting stimulation, individuals need a mechanism to push them out of the vale of monotony into environments that offer more novelty and meaning. Boredom, a common human experience arising broadly throughout all phases of life (Yazzie-Mintz, 2010; Cummings et al., 2016; Finkielsztein, 2023), provides such a mechanism. However, while curiosity has received strong scientific interest since the beginning of modern neurosciences, boredom was widely neglected for a long time, being studied mainly in the context of philosophy (Martin et al., 2006). In the last few decades, scientific interest in boredom has grown, raising more and more questions about its interplay with curiosity and how both phenomena can be conceptualized. In the following section, we discuss classical definitions of boredom and curiosity, review experimental approaches to address both phenomena, and compare how boredom and curiosity differ in regard to their experience and behavioral implications (Figure 1).

### Characterizing boredom and curiosity

Boredom and curiosity appear most commonly as transient mental *states* in response to particular environmental or internal conditions of an individual. How can those states be characterized?

Curiosity has been generally defined as an inherent motivation to explore one's environment (Berlyne, 1960, 1966; Gottlieb et al., 2013; Kidd and Hayden, 2015; Monosov, 2024). In this context, curiosity-driven actions can be separated into two classes based on how immediately they are linked with external reinforcers: Instrumental curiosity describes the urge to seek information that can be used to obtain rewards on a short timescale, whereas non-instrumental (or intrinsic) curiosity describes the urge to seek information not directly linked with a current reinforcement (Kidd and Hayden, 2015; Bromberg-Martin and Sharot, 2020; Gottlieb, 2023; Monosov, 2024). While classical studies in operant behavioral tasks have focused on the former, instrumental definition of curiosity, recent studies in more naturalistic environments emphasize non-instrumental curiosity as a key driver for behavior (Harlow, 1950; Gottlieb, 2012).

Non-instrumental curiosity assumes an inherent subjective value of information. This inherent value drives individuals to actively engage with their surroundings (Oudeyer and Kaplan, 2007), where the conditions for curiosity-driven engagement result from an interaction between individual traits and environmental opportunities to acquire information (Loewenstein, 1994; Silvia and Kashdan, 2009). One prominent characterization of a motivation to acquire information is that of “*knowledge gaps*” (Loewenstein, 1994)—perceived discrepancies between what is known and unknown—which give rise to a directed search aimed at filling the gap (Berlyne, 1960). Taken together, curiosity can be described as an appetitive, internal signal for an individual to engage with specific components of its environment and seek information to complement existing knowledge about the world.

On the opposite side, boredom has been defined as a cognitive state of wanting but being unable to engage in satisfying activity (Eastwood et al., 2012). Such failures to engage can result from situation-dependent environmental causes or from internal, situation-independent factors impeding the ability of an individual to engage with its environment (Todman, 2003). The factors contributing to the emergence of boredom are multifaceted, ranging from environmental monotony (Raz, 2013; Danckert and Gopal, 2024) over attentional deficits (Kass et al., 2003; Eastwood et al., 2012; Westgate and Wilson, 2018) and lacking agency (Struk et al., 2021; Gorelik and Eastwood, 2023) up to low sense of meaning (van Tilburg and Igou, 2012; Westgate and Wilson, 2018). Under such conditions, boredom is conceptualized as an internal signal indicating that the current environment or action no longer provides satisfactory information, pushing the individual to engage in different, more favorable environments or actions (Bench and Lench, 2013; Kurzban et al., 2013). Thus, boredom can be described as a self-regulatory signal to effectively engage one's own cognitive and behavioral resources in environmental demands (Danckert and Elpidorou, 2023). In a similar way, as hunger signals to an individual that it should seek for nutriment in order to avoid starvation, boredom thus indicates that an individual should seek for stimulation to prevent negative effects on mental health, typically associated with sensory deprivation (Mason and Brady, 2009; Raz, 2013; Das et al., 2015; Sahoo et al., 2021).

### Assessments of boredom and curiosity

From an experimental point of view, different methods have been established to assess boredom and curiosity. In human studies, a classical approach is to evaluate boredom and curiosity based on standardized self-report assessments (see Farmer and Sundberg, 1986; Renner, 2006; Litman, 2008; Kashdan et al., 2009; Fahlman et al., 2013; Vodanovich and Watt, 2016; Kashdan et al., 2018 for examples). These self-report tools quantify boredom and curiosity along multiple specific dimensions of experience. For instance, the *Multidimensional State Boredom Scale*, a common tool to assess boredom, has been devised to cover experience with regard to inattention, arousal, time perception, and disengagement (Fahlman et al., 2013). In contrast, the curiosity-related scale *Curiosity and Exploration Inventory* comprises subdimensions that assess the motivation to actively seek new knowledge and embrace statistical unpredictability in one's environment (Kashdan et al., 2009). While useful, these multidimensional assessments are inherently restricted to human populations, due to their reliance on self-reports. For the same reason, relying on self-reports predisposes measures of boredom and curiosity to subjective response biases (McDonald, 2008; Bauhoff, 2014; Lira et al., 2022), limiting the comparability between individuals.

To overcome some of these limitations and to complement these language-based assessments, different behavioral methods have been established to assess boredom and curiosity. For instance, eye movements (Hoppe et al., 2018) and orienting behavior toward novel stimuli have been used as classical proxies for curiosity (reviewed in Kidd and Hayden, 2015; Gottlieb and Oudeyer, 2018; Monosov, 2024), as well as active engagement with particular information sources in a rich sensory environment (Lydon-Staley et al., 2021). To specifically induce curiosity, experimenters have typically presented subjects with ambiguous stimuli or questions (Kang et al., 2009; Jepma et al., 2012), before quantifying an individual's motivation to obtain information that resolves the ambiguity raised (e.g., measures of willingness to pay for revealing the answer to an intriguing question). In a similar way, boredom has been operationalized as stimulus-seeking during situations of high environmental monotony (Meagher and Mason, 2012; Wilson et al., 2014; Yawata et al., 2023), or as a preference for variable stimulation when being confronted with repetitive alternatives (Geana et al., 2016; Bench and Lench, 2018; Seiler et al., 2022) (e.g., through a decision-making task with choosing options that result in less monotonous outcomes). These behavioral methods offer the strong advantage of being applicable in non-human animals, enabling deeper investigations of the neuronal underpinnings of both phenomena. The main caveat of these methods however is their unspecific reliance on general information-seeking behaviors which, as we argue above, may be influenced by both curiosity and boredom (Meier et al., 2024), precluding the demarcation of both phenomena.

To overcome this issue, a crucial component of behavioral tasks used to assess boredom or curiosity is their initial validation in humans where behavioral and self-report measures could be matched. Such validation procedures should demonstrate that the objective behavioral assessment correlates either within or across participants with the participants' self-reports. Importantly, despite the translational and broad applicability of non-verbal, behavioral assessment tools, real-world outcomes of psychological phenomena have been shown to be better predicted by self-reports (Eisenberg et al., 2019), underlining their importance for the validation of behavioral assessment techniques.

Moreover, while many of the behavioral assessments, mentioned above, have been validated by verbal measurements of either curiosity or boredom, including standardized questionnaires for both constructs provides further advantage, allowing to distinguish, whether an observed information-seeking behavior is driven by boredom or curiosity. For example, a behavioral task that quantifies information-seeking through the frequency of choices of a variable compared to monotonous alternative may be coupled with a curiosity questionnaire and an additional boredom questionnaire. A correlation across participants of curiosity with information-seeking behavior may suggest that behavior in the task is driven by curiosity. Alternatively, showing that information-seeking at a given time point is correlated with the present degree of boredom may suggest that task behavior is driven by boredom. Thus, the combination of established and new assessments for boredom and curiosity could deepen the understanding and explanatory separation of the phenomena and, through enabling animal experiments, allow to investigate their neurocognitive correlates.

### Distinct experiential profiles, but congruent function by promoting information-seeking

As commonly encountered in daily life, the experiential correlates of boredom and curiosity are widely oppositional (Danckert and Eastwood, 2020) (Figure 1). On an affective level, boredom is linked with strong negative affect and avoidance of the boredom source (Smith, 1981; Elpidorou, 2018), whereas curiosity is characterized by positive affect and attraction toward a specific information source (Loewenstein, 1994; Gottlieb et al., 2013). This contrast is also reflected on an attentional level, where boredom involves low, drifting attention (Kass et al., 2003; Eastwood et al., 2012; Gerritsen et al., 2014; Westgate and Wilson, 2018), whereas curiosity comes along with high, focused attention (Kinney and Kagan, 1976; Loewenstein, 1994; Gottlieb, 2012; Wojtowicz and Loewenstein, 2020). Regarding the perception of time in both states, boredom is characterized by experiencing time as prolonged and decelerated (Watt, 1991; Zakay, 2014; Witowska et al., 2020; Martarelli et al., 2024), whereas curiosity was linked with flow experience and high cognitive engagement (Schutte and Malouff, 2020) both associated with accelerated, shortened time perception (Csikszentmihalyi et al., 2005; Witowska and Zajenkowski, 2021). These wide differences between boredom and curiosity are less pronounced on the dimension of arousal. While curiosity is linked with high physiological arousal (Berlyne, 1960, 1966; Chatterton, 2022; Gustafsson et al., 2024), boredom is characterized as a state of mixed arousal (Eastwood et al., 2012; Goetz et al., 2014; Merrifield and Danckert, 2014; Raffaelli et al., 2017; Danckert et al., 2018), in which individuals report both, feeling agitated and empty at the same time (Fahlman et al., 2013). This shared aspect between boredom and curiosity of feeling restless, underlines that both phenomena implicate behavioral responses addressed to actively mitigate curiosity or boredom respectively. In the case of boredom, the behavioral response would be to escape the current situation of monotony and search for an alternative environment that provides satisfying stimulation, and thus overcomes boredom (Bench and Lench, 2013; Elpidorou, 2020). Curiosity, on the other hand, would result in the behavioral search for stimulation that can fill and satisfy internal information gaps (Loewenstein, 1994; Litman et al., 2005). Thus, despite the vast dissimilarity in the experience of boredom and curiosity, they both converge on a functional level, promoting the search for novel information that can improve the own knowledge about the world (Henderson and Moore, 1980; Molinaro et al., 2023).

### The interplay of boredom and curiosity

We describe curiosity and boredom as two independent cognitive states, that converge on a functional level. Surprisingly, despite their functional relatedness, both constructs have mostly been studied in isolation, leading to a fragmented understanding of their interplay. Can both states co-occur, or are they mutually exclusive?

We focus our analyses above on the *state* aspects of boredom and curiosity, defining them as transient conditions that only temporally affect cognition and behavior (Loewenstein, 1994; Eastwood et al., 2012). However, it has also been proposed for both constructs that they appear as *traits*, describing general and temporally lasting tendencies to experience boredom or curiosity (Naylor, 1981; Farmer and Sundberg, 1986; Boyle, 1989; Mercer-Lynn et al., 2014). Given the highly distinct experiential profiles of boredom and curiosity, both phenomena as states can be regarded as mutually exclusive, meaning that a person who feels bored in a given moment cannot at the same time feel curious, or vice versa. This exclusiveness of both states is illustrated when considering for instance their dissimilarity in affect: Positive affect would be specific for curiosity while at the same time precluding boredom, whereas negative affect due to boredom would preclude curiosity.

In contrast to their nature as a state, boredom and curiosity on a trait level can co-exist and even influence each other (Hunter et al., 2016). In this context, it was found in self-report-based studies that trait boredom and trait curiosity can exhibit a negative correlation (Eren and Coskun, 2016) as well as a positive correlation (Hunter et al., 2016; Sung et al., 2020), reflected by correlation coefficients in the range of ~-0.3 and +0.5. This opens the possibility for individuals to profit from both, either boredom or curiosity that can both be potentially elicited, depending on the current situational condition. While a highly boredom-prone subject in a given environment, offering average degrees of stimulation, might be overall more likely to experience boredom, the evoked state of boredom can transition into curiosity in the next moment, for instance when changing the environment. Over longer time scales, both states might hence alternate and, even if they do not co-exist at the same time, affect and complement each other over consecutive points in time. In line with this perspective, it was shown that the trait of being open to novel experiences is a strong positive predictor of trait curiosity, but correlates negatively with boredom proneness (Hunter et al., 2016). In the same study, personality traits were identified as significant factors mediating the relationship between boredom and curiosity. Thus, trait boredom and trait curiosity together with other personality features may set a foundation for individual cognitive response tendencies, while at the same time allowing individuals to fall into different states based on the conditions of the current situation (Hunter et al., 2016; Stanek and Ones, 2023), thus flexibly switching between curiosity and boredom.

## Boredom and curiosity from an information-seeking perspective

From a functional viewpoint, curiosity and boredom are drivers of meaningful interaction with an environment. A priori, an organism's energy-efficient policy may imply idle existence. However, the ability to actively engage with one's environment, process the sensory information it provides, and integrate it into our internal model of the world is a fundamental feature of the brain, allowing for flexible and adaptive behavior (Friston, 2010; Summerfield and de Lange, 2014; Behrens et al., 2018). In this context, mechanisms to actively seek information are thought to be essential to learning, expanding individual knowledge, and maximizing the reward obtained from an environment (Gottlieb, 2012; Gottlieb et al., 2013; Gottlieb and Oudeyer, 2018; Monosov, 2024).

Boredom and curiosity both constitute central drivers of information-seeking that arise in different contexts, with distinct pull-push dynamics. In this section, we detail information-oriented perspectives on both phenomena: boredom, on the one hand, pushing individuals away from monotonous information sources (Eastwood et al., 2012; Bench and Lench, 2013; Elpidorou, 2020; Danckert and Elpidorou, 2023), and curiosity pulling individuals toward specific information sources, aimed at filling information gaps (Loewenstein, 1994; Litman et al., 2005; Silvia and Kashdan, 2009). These distinctions affect downstream information-seeking behavior, determining the type of information sought and, as a result the individual's overall knowledge.

### Quantifying the transmission of information in an experimental context

Information, the abundance or scarcity of it, is a key concept in the emergence of curiosity and boredom. The term, information, is widely used in the cognitive neuroscience literature, albeit with significant variability of its context and meaning (Rathkopf, 2017, 2020), ranging from descriptions of environmental features (Floridi, 2011) to the efficiency of neuronal codes (Borst and Theunissen, 1999; Lewicki, 2002; Simoncelli, 2003; Benjamin et al., 2022). A common operationalization of information in the study of boredom and curiosity is some quantification of sensory inputs that are considered useful for improving fitness in an environment, such as a specific experimental task (Berlyne, 1960; Klapp, 1986; Biederman and Vessel, 2006). For instance, a particular stimulus deviating from an individual's common experience, such as the vocalizations of prey, can be considered informative, as it signals the availability of a novel food reward to a hunting animal. In contrast, a repetitive stimulus that does not correlate with reinforcement or punishment would be considered uninformative for an individual. While intuitive, such categorization of environmental conditions being highly or scarcely informative is limited by the subjectivity of the inferred usefulness, and the difficulty of comparing information, for example from two stimuli, on a continuous scale. In contrast to this assess approach, a quantitative approach to assess information may be attained using the mathematical field of Information Theory.

**Figure 2:**
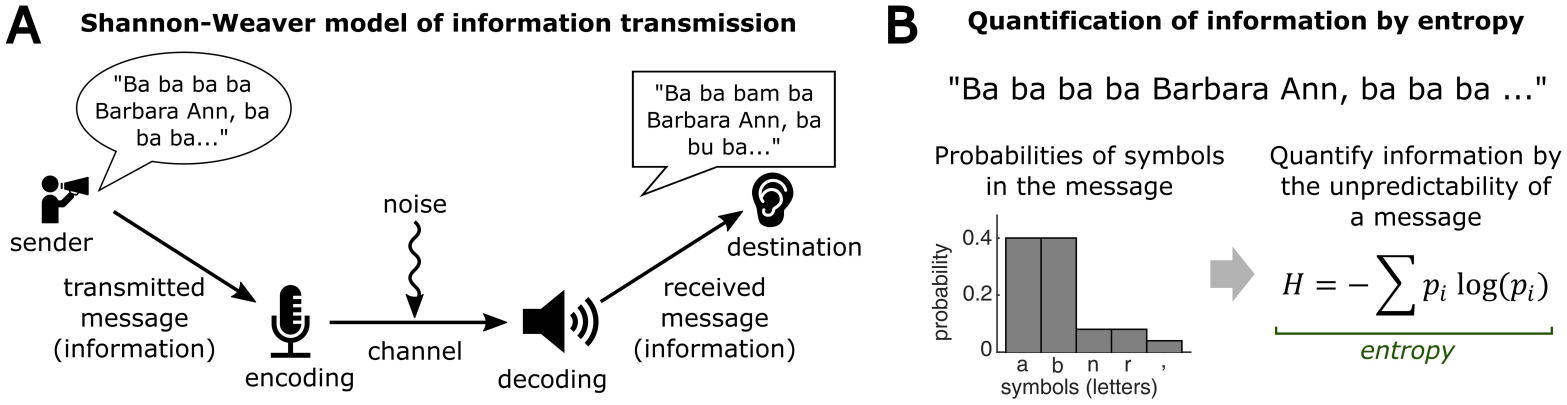
Basic Information Theory allows quantification of information content: **(A)** A standard model of information transmission (Shannon, 1948; Shannon and Weaver, 1949). A message is encoded into a sequence of symbols, and sent out through a communication channel, before becoming decoded and interpreted by a receiver. Thus, effectively transmitted information always depends on the interplay and coherence between sender and receiver. **(B)** Information Theory allows a quantification of the information content of a message as *entropy*, based on the probability distribution of symbols in the message.

Claude Shannon's Information Theory (Shannon, 1948) characterizes a directed flow of information (Shannon and Weaver, 1949) in the form of a message that is (i) sent, (ii) encoded, (iii) passed, through a communication channel, (iv) decoded, and finally (v) received (Figure 2A). In this framework, the effective transmission of information depends not only on the information sent out, but also on the ability of a receiver to decode the sent message. Thus, the overall transmitted information is limited by both, external factors of the sent information, and internal factors of information decoding. To formally quantify the information content of a message, the concept of *entropy* summarizes the probability distribution of a message, providing a metric for the encodability, or “surprise”, of the message. For example, a message with the same letter repeated 20 times would have high encodability, low surprise, and thus low entropy. In contrast, a message with 20 distinct letters would be characterized by high levels of surprise, reflected by a uniform probability distribution, and high entropy (Figure 2B). This quantitative concept of surprise has been shown to be a major factor in neural responses associated with information-seeking, useful for individuals to estimate the degree of noise in particular environments (Monosov, 2024).

The Information Theory framework and the quantitative characterization of an environment's entropy can be broadly applicable to experimental designs, where transmitted information may be conceived as originating from a source (e.g., an environmental auditory stimulus), passing through a channel of a sensory organ (e.g., cochlea) and being decoded by the brain into an updated perception. A quantitative description of information content can provide valuable insights into the neuronal and behavioral responses to different degrees of environmental complexity (Calhoun et al., 2014; Rubin et al., 2016; Lu et al., 2019; Lavdas and Schirpke, 2020; Seiler et al., 2022). An important note on quantifying informational entropy relates to the decision on the level of describing a stimulus, for example, the phonetic vs. semantic content of a spoken message. Here, an individual's capability to effectively decode information from the sensory variability in a message is crucial. In particular, the internal personality features and cognitive abilities of an individual may determine its ability to effectively process sensory stimuli, thus affecting how much information is drawn from a sensory message. Hence, effective information transmission depends on two factors, (i) the amount of objective variability in a sent message, and (ii) the subjective ability of a receiving agent to process the sensory variability and decode the message. This information-theoretic perspective aligns well with the idea that the brain aims to optimally allocate its mental resources to environmental stimulus sources, in order to maximize its cognitive engagement (Danckert and Elpidorou, 2023): A mismatch of the objective information content provided by the current sensory environment and the internal decoding abilities of an agent would lead to low information transmission, reflected by low cognitive engagement. In contrast, a match of the objective information content in the current environment and the internal decoding abilities of an agent would lead to high information transmission, reflected by strong cognitive engagement. Thus, efficient allocation of mental resources to adequate environmental stimuli may drive cognitive engagement by ensuring a continuous, effective transmission of sensory information to the brain.

In summary, the quantitative concepts provided by Information Theory constitute a powerful tool for experimental designs aimed to study information-seeking behaviors. In the context of experimental tasks for the study of boredom and curiosity, the concept of entropy may be used to describe environmental conditions parametrically that are causally associated with both phenomena. Such a quantitative characterization may facilitate the investigation of the computational functions of boredom and curiosity for cognition and behavior in different sensory contexts.

### Effects of boredom and curiosity on information acquisition

The theoretical foundations for quantifying information provide a tool to accurately assess the environmental conditions associated with the emergence of curiosity and boredom. In doing so, these theories allow to study the conditions in which boredom and curiosity emerge in more detail. How do the environmental triggers that give rise to boredom and curiosity differ?

In classical studies, boredom has been shown to arise predominantly in situations of low environmental stimulation, for instance when sitting in an empty room (Wilson et al., 2014; Havermans et al., 2015; Nederkoorn et al., 2016), watching repetitive image sequences (Merrifield and Danckert, 2014; Danckert and Merrifield, 2016; Bench and Lench, 2018; Seiler et al., 2022), or fulfilling monotonous motor actions (Abramson and Stinson, 1977; Markey et al., 2014). Additionally, over-complex environments with high degrees of randomness and unpredictable stimulation have also been shown to elicit boredom (Klapp, 1986; Struk et al., 2021; Danckert and Elpidorou, 2023). Integrating environments with very low and very high stimulus variability into a unified cause of boredom may seem unintuitive. However, an information-theoretic perspective allows bridging both types of environments, characterizing them by low information, a condition that is known to elicit boredom (Klapp, 1986; Seiler et al., 2022; Danckert and Gopal, 2024). While monotonous environments provide low degrees of information by limiting the amount of information in the sensory stimulation *per se*, highly random environments yield stimulation that cannot be effectively decoded and interpreted, thus constraining the effectively received information. Thus, the degree of information in a given situation depends on two factors: (i) the overall unpredictability or entropy of an environment, determining the potential information content of a sent sensory message, and (ii) the ability of an individual to decode and receive the information from this sensory message. In this context, a noise-like sensory input would be highly unpredictable, thus providing high entropy, however, it would be hard for a subject to decode information from such a stimulus, as the receiver lacks an internal framework to interpret the variability in the message and decode it to update its own priors.

In line with this aspect, additional to the environmental factors that contribute to the onset of boredom, a wide range of individual factors are also linked to its emergence. For instance, boredom susceptibility is affected by the degree of a person's agency (Raffaelli et al., 2017; Gorelik and Eastwood, 2023), their attentional capabilities (Kass et al., 2003; Eastwood et al., 2012; Malkovsky et al., 2012; Pironti et al., 2016), and the meaning and sense they attribute to the current activity (van Tilburg and Igou, 2012, 2017; Westgate and Wilson, 2018). As detailed above, from an information-theoretic perspective, factors associated with attention, agency, or the perceived sense of meaning may be linked with decoding abilities, and hence determine the overall information flow (Shannon and Weaver, 1949). Indeed, prior studies have singled out information processing as a major determinant in the emergence of boredom (Jiang et al., 2009; Ghanizadeh, 2011), whereas prior knowledge networks (Phelps et al., 2012; Zhou et al., 2020; Stella et al., 2024), describing the cumulated amount of acquired memories and stored information about the world, are thought to play only a minor role (Bench and Lench, 2013; Kurzban et al., 2013).

**Figure 3:**
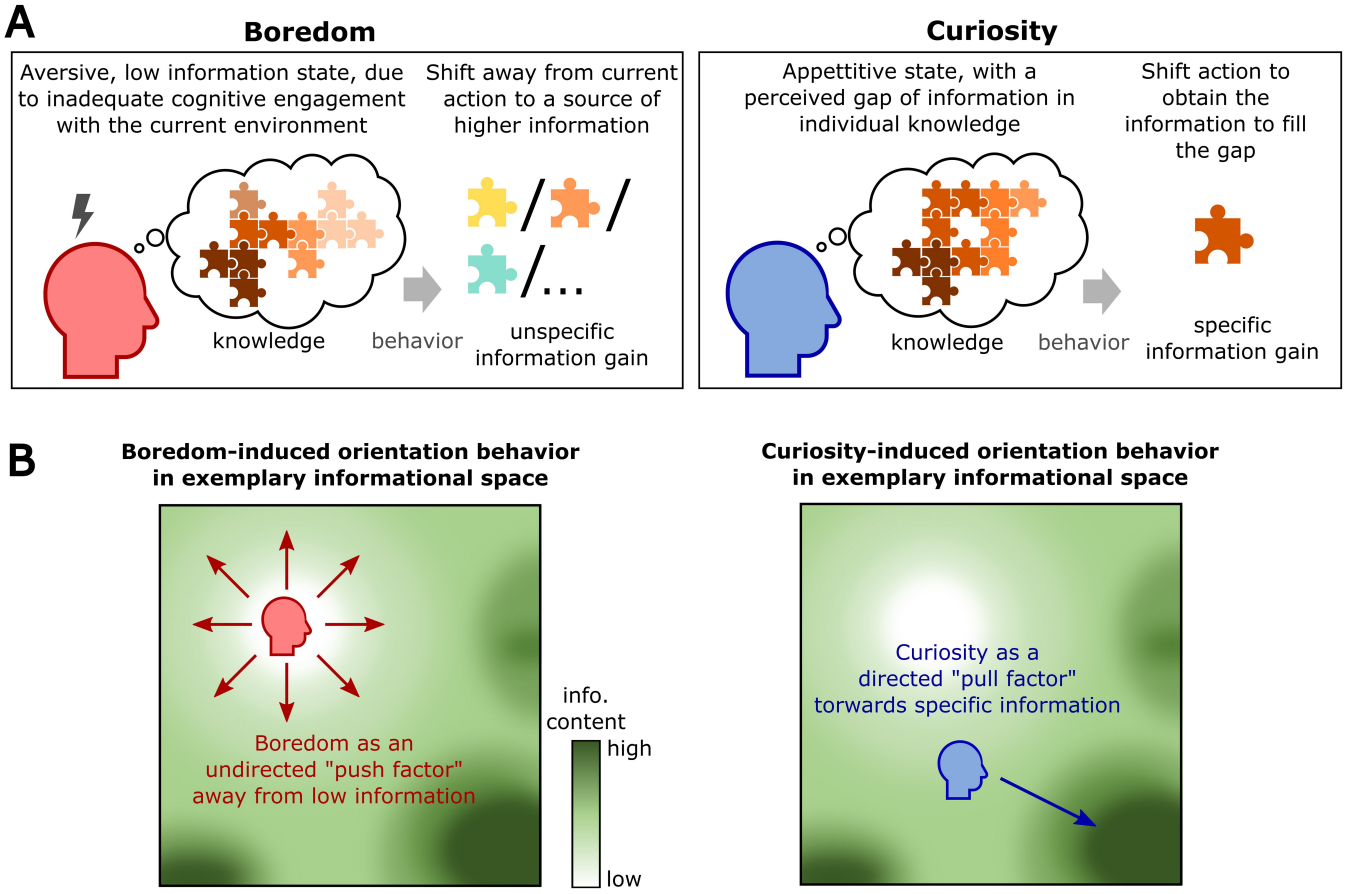
Specific effects of boredom and curiosity on information-seeking: **(A)** Boredom and curiosity differently affect information-seeking. Left: Boredom arises in states of low information transmission, largely independent from prior knowledge, and unspecifically shifts behavior toward other sources of information in the environment. Right: Curiosity arises from specific gaps in the knowledge of an individual, largely independent from current information transmission, and drives behavior to fill these knowledge gaps by acquiring specific pieces of information. **(B)** Illustration of action trajectories of bored and curious agents in an exemplary environment that offers sources with varying information content. Left: Boredom would push individuals away from sources of monotony (low information) to unspecifically explore sources of higher information. Right: In contrast, curiosity would pull individuals to exploit specific sources of information that fill internal information gaps.

Taken together, boredom can be characterized as an internal signal to indicate states of low information, driving agents toward stimulus sources that provide higher information (Klapp, 1986; Geana et al., 2016; Danckert and Gopal, 2024) (Figure 3A left). This undirected drive of boredom on behavior is highlighted in multiple studies, reporting that boredom can drive any type of behavior, independent of whether it leads to positive or negative consequences. For instance, subjects were found to even actively seek unpleasant sensory stimuli when being bored, such as emotionally aversive pictures (Bench and Lench, 2018) or electric shocks (Wilson et al., 2014; Havermans et al., 2015; Nederkoorn et al., 2016). In addition, boredom was more broadly linked to a variety of qualitatively diverging behaviors, ranging from positive, adaptive behaviors (Bench and Lench, 2013; Bieleke et al., 2022; Seiler and Rumpel, 2023) and creativity (Mann and Cadman, 2014; Gomez-Ramirez and Costa, 2017), up to maladaptive behaviors such as gambling (Blaszczynski et al., 1990; Fortune and Goodie, 2010; Bonnaire and Barrault, 2018), substance abuse (Krotava and Todman, 2014; Phillips et al., 2017; Windle and Windle, 2018), rule-breaking (Wolff et al., 2020; Drody et al., 2022) and crime (Heide, 1997). Hence, boredom affects behavior by pushing individuals away from the current source of low information and driving them to seek information in alternative actions in a non-directed manner.

In contrast to boredom, curiosity typically arises independently from the overall environmental information content, and is determined by specific gaps in the knowledge of an individual (Berlyne, 1966; Loewenstein, 1994; Litman et al., 2005; Silvia and Kashdan, 2009). In accordance with this information gap hypothesis, classical tasks to study curiosity have presented subjects with blurred, permuted stimuli or questions to raise specific gaps of information, and then assessed the subjects' sentiment and behavior as they tried to fill these information gaps (Kang et al., 2009; Jepma et al., 2012; Wade and Kidd, 2019). Information gaps constitute lacking knowledge elements that can complement individual knowledge networks and often arise from external stimulation, priming an individual for an unknown bit of information (Loewenstein, 1994). In accordance, curiosity was found to be highest in situations with an intermediate degree of uncertainty, reflecting information gaps that are not trivial, but can still be estimated by individuals (Kang et al., 2009; Dubey and Griffiths, 2020; Spitzer et al., 2024). As curiosity is associated with positive affect and the activation of intrinsic reward systems upon the identification and the filling of a knowledge gap (Kang et al., 2009; Jepma et al., 2012; Gottlieb et al., 2013; Monosov, 2024), it drives individuals to behaviorally seek for the specific piece of information complementing its prior knowledge (Gottlieb, 2012; Wade and Kidd, 2019) (Figure 3A right). In line with this, curiosity has further been identified as a driver for learning (Oudeyer and Kaplan, 2007; Kang et al., 2009; Gruber et al., 2014; Kidd and Hayden, 2015; Gottlieb and Oudeyer, 2018; Poli et al., 2024) and was related to the development of creative solutions (Evans and Jirout, 2023; Ivancovsky et al., 2024), suggesting that achieved information from the curious process is effectively integrated into existing knowledge networks, serving as a basis for novel, innovative associations. Interestingly, it was shown that ratings of curiosity even increase as individuals start to fill their information gaps by achieving the desired sensory input (Litman et al., 2005; van Dijk and Zeelenberg, 2007). This suggests that by raising an information gap, individuals develop an estimate of the information they want to obtain, before then searching their environment to fill this gap. Hence, curiosity constitutes a state that promotes the specific search for information that fits and integrates into empty spots of an individual's knowledge.

Taken together, boredom and curiosity both promote the search for novel information, however, they vary in the type of information they achieve: boredom arises from states of low information, signaling the demand to unspecifically search for any type of information, whereas curiosity arises from defined gaps in internal knowledge, directing individuals to achieve the specific information that complements internal priors.

### Complementing effects on exploration and exploitation behavior

**Figure 4:**
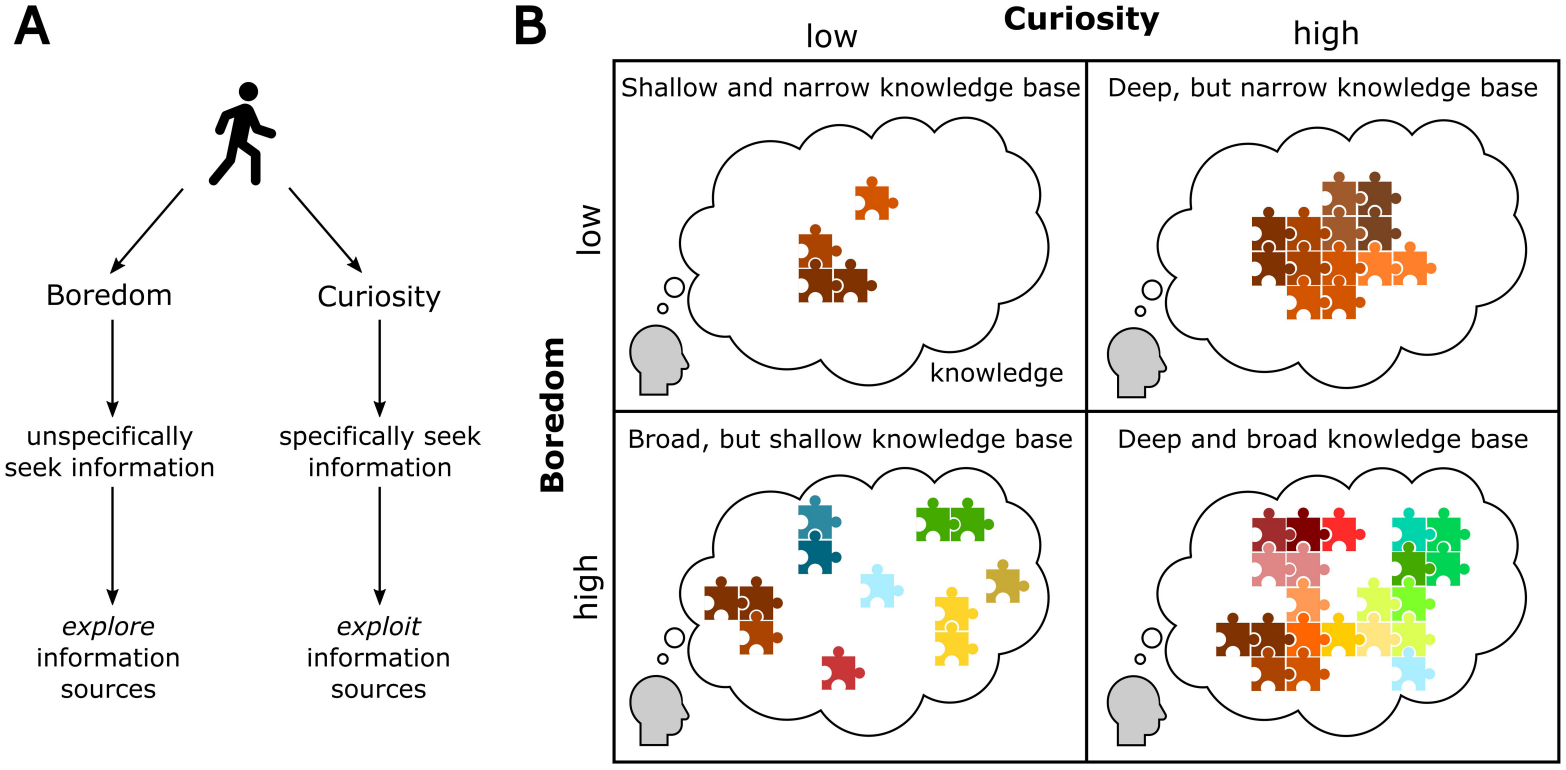
Predicted knowledge structures for individuals with different boredom and curiosity profiles. **(A)** Given the distinct effects on information-seeking (see Figure 2), boredom essentially drives unspecific *exploration* behavior, whereas curiosity drives specific *exploitation* of information sources. **(B)** This predicts different knowledge structures of agents characterized by varying extent of boredom and curiosity respectively. Agents with low boredom and low curiosity would only have narrow and scarcely developed knowledge base. High boredom and low curiosity would lead to a widely spread knowledge network that however shows only low density and hence leaves many gaps. High curiosity and low boredom would lead to a dense knowledge base with only few information gaps, however with only little extent on different topics. High boredom and high curiosity combined would lead to a multidimensional and densely connected knowledge network.

The above characterization of boredom and curiosity as two independent cognitive signals that drive the search for unspecific vs. specific information, highlight both phenomena as functionally complementary cognitive mechanisms (Hunter et al., 2016; Yu et al., 2019; Wojtowicz and Loewenstein, 2020). Specifically, while boredom emerges under selective conditions where an individual's current environment only provides scarce information, curiosity can emerge independently from the current environment, evoked merely by raised information gaps in internal knowledge (Figure 3B). Moreover, boredom pushes individuals away from the current action to find higher information, whereas curiosity attracts individuals to specific sources of information (Noordewier and Gocłowska, 2024). Hence, the functions of boredom and curiosity can be compared to other cognitive signals such as hunger and appetite that serve complementing purposes: Similar to *hunger*, which can be satisfied with basically any food, boredom drives individuals to engage in almost any action that leads to enhanced information. Importantly, in this condition subjects do not need any a-priori estimate of the quality of this achieved information, making boredom a central driver to explore novel actions and environments (Bench and Lench, 2013; Gomez-Ramirez and Costa, 2017; Bench and Lench, 2018; Darling, 2023) (Figure 4A). In contrast, the function of curiosity can be well compared to *appetite*, directing individual behavior toward specific sources of stimulation that can integrate into specific knowledge gaps (Gottlieb, 2012; Kidd and Hayden, 2015; Gottlieb and Oudeyer, 2018). Curiosity by definition requires a vague estimate of the desired information (Loewenstein, 1994; Wade and Kidd, 2019; Spitzer et al., 2024), defined by the individual knowledge network associated to the information gap. This in consequence promotes an exploitation of stimulation sources from which an individual would expect to receive the specific, desired bits of information (Figure 4A). Thus, boredom acts as a safeguard mechanism to prevent individuals from stagnancy in uninformative, meaningless environments (Bench and Lench, 2013; Elpidorou, 2018; Danckert and Elpidorou, 2023; Seiler and Rumpel, 2023), whereas curiosity attracts individuals to develop their knowledge about the world and increase their behavioral fitness (Gottlieb and Oudeyer, 2018; Losecaat Vermeer et al., 2022; Monosov, 2024), appearing in basically any environment. Both features together can serve to balance out exploitation-exploration strategies, overcome local minima of information in an environment and hence optimize long-term reward (Gottlieb et al., 2013; Marvin and Shohamy, 2016; Gomez-Ramirez and Costa, 2017; Yu et al., 2019; Poli et al., 2024).

Imaging studies, investigating the neuronal underpinnings of boredom and curiosity, support this view of a complementing role of both phenomena. Boredom has been linked to increased activity in the default-mode network, a set of brain regions involved in task-unrelated signal processing and mind-wandering (Andrews-Hanna, 2012; Smallwood et al., 2021), and anti-correlated activity in the insular cortex, involved in the detection of salient and behaviorally relevant sensory events (Menon and Uddin, 2010; Danckert and Merrifield, 2016; Dal Mas and Wittmann, 2017; Yawata et al., 2023). These observations suggest that the state of boredom is characterized by disengagement with the current sensory input and a shift toward internally generated neural processes and behavioral re-orientation. Moreover, specific activity patterns in the amygdala could hint at a neural correlate of the negative experience during boredom (Ulrich et al., 2014). In contrast, curiosity involves different brain regions, spanning reward-related areas in the dopaminergic midbrain as well as regions related to memory formation (Kang et al., 2009; Jepma et al., 2012; Gruber et al., 2014). Thus, curiosity employs specific networks specialized to achieve novel information and integrate it into memory in order to reduce uncertainty about the world (Monosov, 2024).

On a behavioral level, a recent study (Lydon-Staley et al., 2021), investigating individual exploration trajectories of human participants in an online encyclopedia under unconstrained conditions, revealed that humans fall into two distinct types of information-seeking, aligning with the functions of boredom and curiosity. Either subjects browsed the encyclopedia in a broad and seemingly erratic way, jumping between highly dissimilar articles, or subjects predominantly explored related and inter-connected articles of the encyclopedia. According to the above characterizations, the former, explorative behavior would be attributed to higher degrees of boredom, whereas the latter, exploitative behavior would correspond to high degrees of curiosity in a subject. While real behavior of individuals over longer time periods will likely be affected by both, boredom and curiosity, this dichotomic segregation illustrates that boredom and curiosity result in different consequences on individual knowledge networks. By filling information gaps, curiosity fosters densely interconnected knowledge networks. Boredom, on the other side, by driving unspecific exploration, would lead to wide, but sparsely connected knowledge networks. Together, boredom and curiosity thus enable adaptive information-seeking strategies, optimizing exploration and exploitation behavior to the informational landscape of a given environment and enabling an efficient development of individual knowledge in breadth and depth. The knowledge structures, achieved by both processes would set a foundation for various cognitive processes that may operate on stored knowledge and recombine it to enable intelligent and adaptive behavior (Berg and Sternberg, 1985).

## Directions in future research

In this review, we compare boredom and curiosity on an experiential and functional level, defining its specific consequences in respect to information-seeking behavior. These delineations allow predictions about particular types of behavior and knowledge structure in individuals whose behavior is dominated by boredom or curiosity respectively. We outline these hypotheses in this section, providing an outlook for potential future studies to expand the understanding of the interplay between boredom and curiosity. Furthermore, we discuss potential approaches to deepen the understanding of intrinsic information-related factors that contribute to the emergence of boredom and curiosity, as well as implications of our review for clinical conditions.

### Testing the consequences of different boredom and curiosity profiles on individual knowledge

As portrayed above, boredom exerts an unspecific and undirected drive on behavior, promoting exploration of unknown information sources, whereas curiosity attracts individuals to exploit specific information-sources to fill internal knowledge gaps (Figure 4A). Interestingly, these complementary functions together with the assumption that boredom and curiosity can co-occur in individuals as temporally stable traits, setting different likelihoods for an individual to experience either boredom or curiosity in a given situation, predict characteristic developments of individual knowledge networks for subjects that are more prone to boredom or curiosity, respectively (Figure 4B). For instance, individuals low in curiosity and boredom proneness would be expected to only show little information-seeking, likely resulting in anxious behavior and scarce knowledge bases. In contrast, individuals with high boredom but low curiosity would tend to develop wide but shallow knowledge, covering various pieces of hardly related information. Individuals with low boredom but high curiosity would contrarily develop densely interconnected knowledge networks, which however are confined to only a low range, i.e., detailed knowledge which however does not cover many topics. As a last condition, high boredom proneness combined with high curiosity would drive individuals to develop wide-ranging and well interconnected knowledge.

While these hypothetical scenarios only depict schematic extremes on which individuals could map, they can still be useful to generate intuitions about how different constellations of boredom and curiosity could relate to knowledge structures, thus constituting fundamental factors that broadly influence cognitive function (Biederman et al., 1982; Friston, 2005; Summerfield and de Lange, 2014). To address these hypotheses, future work could measure boredom and curiosity proneness in individuals and then try to assess their knowledge base (Zhou et al., 2020). For instance, knowledge structures in the semantic domain have been successfully assessed by asking individuals to rate the relatedness of multiple different word pairs (Kenett et al., 2014; Benedek et al., 2017; Beaty and Kenett, 2023), correlating to the associative skills of an individual. Adopting similar strategies and using them in the context of boredom and curiosity could link both constructs with individual architectures of associative networks, thus building a bridge to higher cognitive functions (Lydon-Staley et al., 2021). Moreover, in an educational setting, specific interventions to enhance boredom or curiosity could help learners to systematically expand individual knowledge in breadth or in depth (Jirout et al., 2018; Bekker et al., 2023; Bjerknes et al., 2024; Jirout et al., 2024).

### Addressing the effects of internally generated information on boredom and curiosity

In this review, we discuss boredom and curiosity from an information-centric perspective, allowing a systematic comparison of their functional implications. In order to practically apply this concept and translate it into experiments, systematic frameworks to quantify environmental information are required. As we discuss in this review, Information Theory provides a strong tool to pursue this aim, allowing for instance to describe the objective information content of a message by its empirical entropy. However, besides environmental information, individuals have also been shown to draw significant stimulation from internal processes, such as mind-wandering or day-dreaming (Christoff et al., 2016; Danckert, 2017; Martarelli et al., 2020). These internal sources of information are currently hardly captured by most experimental paradigms to study information-seeking. Thus, even if entropy allows to estimate the amount of information provided by presented external stimuli in an experiment, additional factors are needed to quantify the amount of information an individual draws from internal mental processes like spontaneous thoughts and associations. To address this issue and systematically delineate the factors of external and internal information-seeking, a general framework to conceptualize intrinsic and extrinsic information on a neuronal level would be valuable. One candidate approach useful in this context could be provided by *representational similarity analysis* (Kriegeskorte et al., 2008; Diedrichsen and Kriegeskorte, 2017; Noda et al., 2024). Here, specific patterns of brain activity, temporally locked to certain external or internal events, are compared against each other to construct a complex map of similarities between neuronal event representations (Kriegeskorte et al., 2008; Schütt et al., 2023). Implicitly, the distances between represented events provide a measure of their relatedness, where surprising, and thus presumably highly informative, events would be expected to stand out prominently from other represented elements (Li, 2002; Rubin et al., 2016). Combined with behavioral and psychometric assessments, such neuronal metrics could in the future enable to investigate how curiosity and boredom are affected by internal thought processes, and how they evolve over time.

### Assessing boredom and curiosity under clinical conditions

A variety of clinical studies have demonstrated a substantial link of boredom and curiosity to the mental health status of individuals. For instance, while curiosity has been identified as a protective factor of mental health (Sakaki et al., 2018; Gruber and Ranganath, 2019; Losecaat Vermeer et al., 2022), even correlating with reduced overall mortality (Swan and Carmelli, 1996), boredom was linked to a wide range of psychopathologies (Todman, 2003; Todman et al., 2008; Goldberg and Danckert, 2013; Marshall et al., 2019; Seiler et al., 2023) and mental distress (Droit-Volet et al., 2020; Wolff et al., 2020; Danckert, 2022). Moreover, it is well established that dysbalanced motivation to seek information can result in extreme risk affinity and malfunctional behavior (Zuckerman, 1990). Despite these relevant implications, it remains unclear whether boredom and curiosity *per se* are associated with a distinct mental health status, hence constituting an inherent correlate of the psychopathological syndrome, or if both states are actually precursors of changes in mental health.

In this context, it would be interesting to specifically induce one of the two cognitive states, boredom or curiosity, and then assess the other one while also measuring effects on mental wellbeing. Such investigations could provide a detailed perspective on shared dimensions between both states and their relations to other cognitive processes involved in mental health (Hunter et al., 2016; Noordewier and Gocłowska, 2024).

Furthermore, longitudinal assessments of boredom and curiosity together with mental health parameters in diverse cohorts will in the future be helpful to unravel the clinical association of both phenomena. In particular, such studies could help to identify specific vulnerable periods in which individuals, prone to develop psychopathology, could be supported by enforcing curious processes and hence achieve balanced information-seeking strategies. In this context, curiosity could be a major factor that enhances an individual's ability to experience flow (Schutte and Malouff, 2020), a state linked with highly effective problem-solving and internal satisfaction (Csikszentmihalyi et al., 2005) as well as higher mental resilience (Gaston et al., 2024). Thus, clinical outcomes could be improved by a better understanding of the interplay between boredom and curiosity under healthy, preclinical and pathological conditions.

## Conclusion

Boredom and curiosity constitute highly relevant cognitive states, ubiquitously experienced across life. While both phenomena are characterized by distinct and widely opposing experience, they functionally cooperate to drive information-seeking. Boredom, characterized by negative affect, low attention and prolonged time perception, typically arises in situations of low sensory information transmission to an individual and drives individuals to behaviorally search for any source of higher information. Curiosity, defined by positive affect, enhanced attention and shortened time perception, arises from defined internal knowledge deficits, driving individuals to seek for specific information to fill these deficits. Hence boredom and curiosity form a functional unit, complementing each other: Similar to hunger, boredom pushes individuals to unspecifically explore, whereas curiosity, similar to appetite, pulls individuals to exploit specific information sources. These cooperative effects on different dimensions of information-seeking guide individuals to flexibly adjust their behavior in environments with varying and dynamic sources of sensory information. Considering this interplay can in the future help to delineate boredom- and curiosity-related effects in empirical studies, and hence yield a deeper understanding of how both phenomena are rooted in the brain and how they affect cognition.
